# Antimicrobial peptide FF/CAP18 induces apoptotic cell death in HCT116 colon cancer cells via changes in the metabolic profile

**DOI:** 10.3892/ijo.2015.2887

**Published:** 2015-02-10

**Authors:** KENGO KURODA, TOMOKAZU FUKUDA, HIROSHI ISOGAI, KAZUHIKO OKUMURA, MARIJA KRSTIC-DEMONACOS, EMIKO ISOGAI

**Affiliations:** 1 Graduate School of Agricultural Science, Tohoku University, Aoba-ku, Sendai 981-8555, Japan; 2 Animal Research Center, Sapporo Medical University, Sapporo 060-8556, Japan; 3 Department of Oral and Maxillofacial Surgery, School of Dentistry, Health Sciences University of Hokkaido, Hokkaido 061-0293, Japan; 4 School of Environment and Life Sciences, College of Science and Technology, Cockcroft Building, University of Salford, Salford M5 4WT, UK

**Keywords:** HCT116 cell line, antimicrobial peptide, apoptosis, metabolome analysis, CE-TOFMS

## Abstract

Metabolic reprogramming is one of the hallmarks of cancer and can be targeted by therapeutic agents. We previously reported that cathelicidin-related or modified antimicrobial peptides, such as FF/CAP18, have antiproliferative effects on the squamous cell carcinoma cell line SAS-H1, and the colon carcinoma cell line HCT116. Although antimicrobial peptides have potential use in the development of new therapeutic strategies, their effects on the metabolism of cancer cells are poorly understood. Here, we investigated changes in the levels of metabolites in HCT116 cells caused by FF/CAP18, via capillary electrophoresis time-of-flight mass spectrometry (CE-TOFMS). Analysis of the 177 intracellular metabolites and 113 metabolites in conditioned medium that were detected by CE-TOFMS, revealed dramatic changes in the metabolic profile of HCT116 cells after treatment with FF/CAP18. The metabolic profile showed that the levels of most metabolites in the major metabolic pathways supported the rapid proliferation of cancer cells. Purine metabolism, glycolysis, and the TCA cycle, were altered in FF/CAP18-treated cells in a dose-dependent manner. Our present study provides mechanistic insights into the anticancer effects of antimicrobial peptides that show great potential as new therapies for colon cancer.

## Introduction

Cancer is described as one of the major world health problems (1). Colorectal cancer is the third most common malignancy worldwide and nearly 1.4 million new cases were reported in 2012 (2). Accumulation of oncogenes and tumor suppressor gene mutations can contribute to cancer development. High-throughput DNA sequencing data suggested that thousands of point mutations, translocations, amplifications, and deletions may contribute to cancer development, and that the mutational range can differ, even among tumors with identical histopathology (3). Therefore, any therapeutic strategy designed to target individual signaling molecules has limitations in improving current survival rates and novel strategies are needed.

Metabolic reprogramming is one of the hallmarks of cancer, in addition to gene mutation (4). To support rapid cell division and the process of tumor progression, cancer cells need to generate energy by reprogramming their metabolism. It is well established that cancer cells can generate ATP through glycolysis rather than oxidative phosphorylation, even in the presence of oxygen (the Warburg effect) (5). Characteristic metabolic reprogramming, including the Warburg effect, is consistently seen in various cancers, despite numerous gene mutations, indicating that cancer cell metabolic pathways could be useful therapeutic targets. Hirayama *et al* (6) and Soga *et al* (7) reported metabolic profiling of human colon and stomach cancers, and compared the levels of metabolites in tumor and normal tissues using capillary electrophoresis time-of-flight mass spectrometry (CE-TOFMS). Recently, the use of metabolome analysis has remarkably developed in various research fields, such as clinical research, cell biology, and plant studies (8–10). Metabolomics is the final step in the ‘omics’ cascade, of genomics, transcriptomics, and proteomics, and can provide global information on low-molecular-weight-metabolites (11,12). Metabolome analysis could reveal the influences on cancer metabolism of anticancer agents, and accelerate biomarker discovery based on the determination of metabolomic differences between normal and cancerous tissue.

Members of the cathelicidin family of antimicrobial peptides are endogenous factors playing key roles in cancer regulation (13). Human cathelicidin antimicrobial protein, hCAP18, is the only member of the cathelicidin family in human cells; its C-terminal domain, LL-37, is released by proteolytic cleavage, and shows various effects, such as antibacterial, antiviral, wound-healing, and immunoregulatory effects (14,15). LL-37 is expressed in epithelial cells of a number of organs (16). A previous study showed that the expression of LL-37 was markedly downregulated in human colon cancer tissue, whereas exogenous LL-37 induced apoptotic cell death in cultured colon cancer cells. In addition, cathelicidin-deficient mice exhibited increased susceptibility to azoxymethane-induced colon carcinogenesis (17).

We previously reported that a 27-residue analog of the LL-37 peptide, FF/CAP18, induced apoptotic cell death, via mitochondrial membrane depolarization and DNA fragmentation, in the oral squamous cell carcinoma cell line SAS-H1, (18) and the colon carcinoma cell line HCT116 (19). Although these findings suggest that antimicrobial peptides have possible anticancer effects and could be targeted for new therapeutic strategies, the full mechanisms of their suppressive effects on metabolic pathways are still largely unknown. In the present study, using metabolome analysis by CE-TOFMS, we identified changes in energy metabolism caused by FF/CAP18 during the process of apoptosis in human colon cancer cells.

## Materials and methods

### Cell culture and peptides

The human HCT116 colon carcinoma-derived cell line was provided by Dr Bert Vogelstein (Johns Hopkins University, Baltimore, MD, USA). The cells were maintained in Dulbecco’s modified Eagle’s medium (Nacalai Tesque, Kyoto, Japan) containing 10% fetal bovine serum (Invitrogen, Carlsbad, CA, USA) and a 5% antibiotic-antimycotic mixed stock solution (Nacalai Tesque) at 37°C and 5% CO_2_. Before being used for experiments, cells were routinely maintained under exponential-proliferation conditions. The cells were treated with a 0.25% trypsin-EDTA solution (Nacalai Tesque) to dislodge them at each passage.

The primary structure of LL-37 is represented in a single amino acid code as follows: LLGDFFRKSKEKIGKEFKRIV QRIKDFLRNLVPRTES. To enhance antimicrobial activity, FF/CAP18 was designed by the replacement of a glutamic acid residue and a lysine residue with phenylalanine at positions 11 and 20, respectively, of the 27mer (FRKSKEKIGKEFKRI VQRIKDFLRNLV) which resulted from the removal of the first and last five amino acids of LL-37 (20). FF/CAP18 (FRKS KEKIGKFFKRIVQRIFDFLRNLV) was synthesized by the method previously described (18).

### Detection of apoptosis using a combined Annexin V-7-amino-actinomycin D (7-AAD) assay

One feature of the early stages of apoptosis is externalization of plasma membrane phosphatidylserine to the cell surface. Owing to this process, cells showing the early stages of apoptosis can be identified via binding of Annexin V, which has high affinity for phosphatidylserine, whereas cells in the late stage of apoptosis or necrosis show no affinity for Annexin V. Furthermore, 7-AAD, a fluorescent DNA-binding agent that intercalates between cytosine and guanine, also allows the distinction of cells that are alive, dead, or in the early or late stages of apoptosis. The combination of these two reagents is available as a powerful apoptosis-detection tool in the Muse^®^ Annexin V and Dead Cell assay kit (Merck Millipore, Darmstadt, Germany). After incubation with FF/CAP18 for 96 h, cells were trypsinized, transferred into 1.5-ml microtubes, and subjected to centrifugation at 800 × g for 5 min. Cell pellets were resuspended in 100 μl of fresh medium, and the Muse Annexin V and Dead Cell Dye assay kit reagent was added (100 μl to each tube) with mixing. After incubation for 20 min at room temperature, cells were applied to a Muse Cell Analyzer (Merck Millipore).

### Metabolome extraction

Samples of cells and of conditioned medium were obtained 96 h after administration of FF/CAP18. Cell samples were washed twice with a 5% solution of mannitol and covered with methanol. Cells were harvested after addition of the internal standard solution (Human Metabolome Technologies, Tsuruoka, Japan), and subjected to centrifugation for 5 min at 2,300 × g, 4°C. The aqueous layers were collected into ultrafiltration units (EMD Millipore, Billerica, MA, USA) and subjected to centrifugation for 2.5 h at 9,600 × g, 4°C. The conditioned medium from cell cultures was directly collected to prepare medium samples. The sampled medium was mixed with the internal standard solution, and subjected to centrifugation for 2.5 h at 9,600 × g, 4°C.

### Measurement of metabolites

CE-TOFMS was carried out using an Agilent 7100 CE system equipped with an Agilent 6210 TOFMS system, Agilent 1100 high-performance liquid chromatography system with isocratic pump, Agilent G1603A CE-Mass Spectrometry (MS) Adapter kit, and Agilent G1607A CE Electrospray Ionization-MS Sprayer kit (Agilent Technologies, Waldbronn, Germany). The systems were controlled using the Agilent G2201AA ChemStation software, version B.03.01, for CE (Agilent Technologies). Metabolites were analyzed using a fused silica capillary (50 μm internal diameter × 80 cm length), with commercial electrophoresis buffer (Solution ID: H3301-1001 for cation analysis and I3302-1023 for anion analysis; Human Metabolome Technologies) as the electrolyte. The sample was injected at a pressure of 50 mbar for 10 sec (equivalent to ~10 nl) in the cation analysis, and at 50 mbar for 25 sec (equivalent to ~25 nl) in the anion analysis. The spectrometer was scanned across a mass-to-charge ratio (m/z) of 50–1,000. Other conditions were as previously described (21–23).

The peaks detected by CE-TOFMS were extracted using MasterHands automatic integration software (Keio University, Tsuruoka, Japan) in order to obtain peak information including m/z, migration time (MT), and peak area (24). Signal peaks corresponding to isotopomers, adduct ions, and other product ions of known metabolites were excluded, and the remaining peaks were annotated with putative metabolites from the Human Metabolome Technologies metabolite database, based on their MT and m/z values. The tolerance range for the peak annotation was configured at ±0.5 min for MT and ±10 parts per million for m/z. In addition, peak areas were normalized to those of the internal standards, and the resultant relative area values were then further normalized to the sample amount.

Hierarchical cluster analysis (HCA) and principal component analysis (PCA) were carried out using the proprietary software, PeakStat and SampleStat, respectively (Human Metabolome Technologies). Detected metabolites were plotted on metabolic pathway maps using the Visualization and Analysis of Networks containing Experimental Data software (25).

### Statistical analysis

In the combined Annexin V binding-7-AAD staining assay, the ratio of cells at each apoptotic stage was expressed as the mean ± standard deviation. Statistical differences were tested with Student’s t-test. The statistical significance of differences in the 177 intracellular metabolites and the 113 metabolites detected in conditioned medium, between treated and untreated cells, was determined using Welch’s t-test. A value of P<0.05 was considered significant.

## Results

### Apoptosis detection using the Annexin V-7-AAD assay

**Figure 1:**
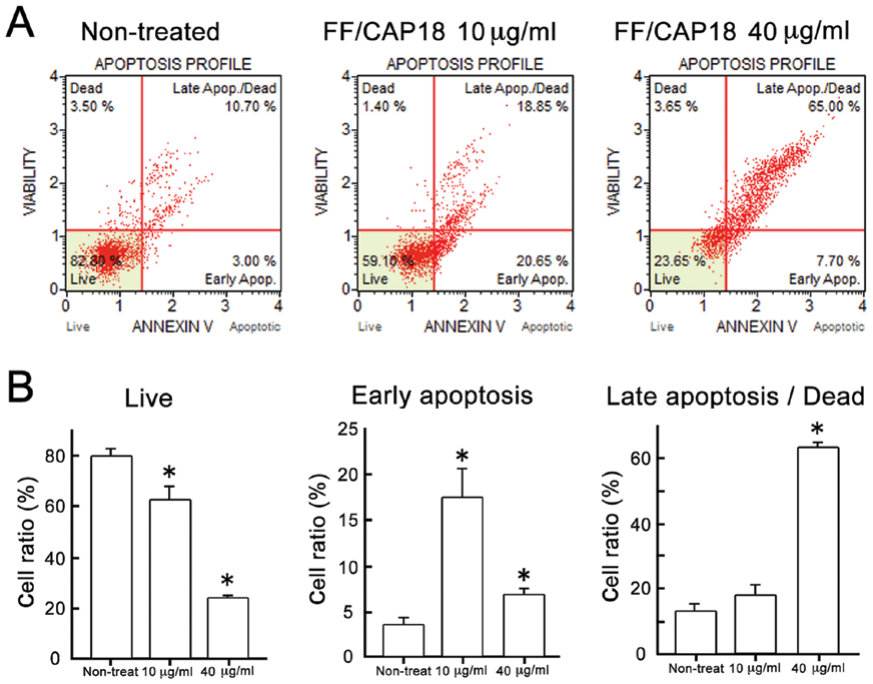
Detection of apoptosis of HCT116 cells after treatment with FF/CAP18. Cells were treated with 10 or 40 μg/ml FF/CAP18 for 96 h and subjected to the combined Annexin V binding-7-AAD staining assay. (A) Representative results of the assay carried out with non-treated HCT116 cells (left panel), and with HCT116 cells treated with FF/CAP18 at 10 μg/ml (middle panel) or 40 μg/ml (right panel). Based on the reactivity with Annexin V and the intensity of the 7-AAD fluorescence, cells can be classified into four categories: dead, live, early apoptosis and late apoptosis/dead. Triplicate experiments were conducted and representative results are shown. (B) The percentage of live cells (left panel), cells in early apoptosis (middle panel) and cells in late apoptosis or dead (right panel). Triplicate samples were used to obtain the mean and standard deviation. The asterisks indicate statistical significance. ^*^P<0.05.

Combined Annexin V and 7-AAD reactivity allowed classification of cells into four groups, as follows: early apoptotic cells [Annexin V (+) and 7-AAD (−)], late apoptotic or dead cells [Annexin V (+) and 7-AAD (+)], dead cells [Annexin V (−) and 7-AAD (+)], and live cells [Annexin V (−) and 7-AAD (−)]; see the scatter plots in Fig. 1A. Treatment of HCT116 cells with FF/CAP18 at 10 μg/ml induced high affinity for Annexin V, as shown by the right shift of the scatter plot compared with that of non-treated cells, indicating early apoptosis (Fig. 1A, middle panel). On the other hand, FF/CAP18 treatment at 40 μg/ml increased the number of cells that were positive for Annexin V (+) and 7-AAD (+), indicating that a high dose of FF/CAP18 induced apoptotic cell death in HCT116 cells. The ratio of HCT116 cells at each stage of apoptosis after treatment with the two different doses of FF/CAP18 is summarized in Fig. 1B. The percentage of live cells decreased significantly in a dose-dependent manner (Fig. 1B). The percentage of cells in early apoptosis, however, significantly increased with 10 μg/ml FF/CAP18 treatment, whereas, the percentage of late apoptotic and dead cells only increased with 40 μg/ml treatment (Fig. 1B). From these results, we concluded that early-stage apoptosis was induced by a comparatively low dose (10 μg/ml) of FF/CAP18, whereas high-dose treatment (40 μg/ml) could cause late-stage apoptosis and cell death.

### Heat map and PCA representation of metabolome data from HCT116 cells

**Figure 2:**
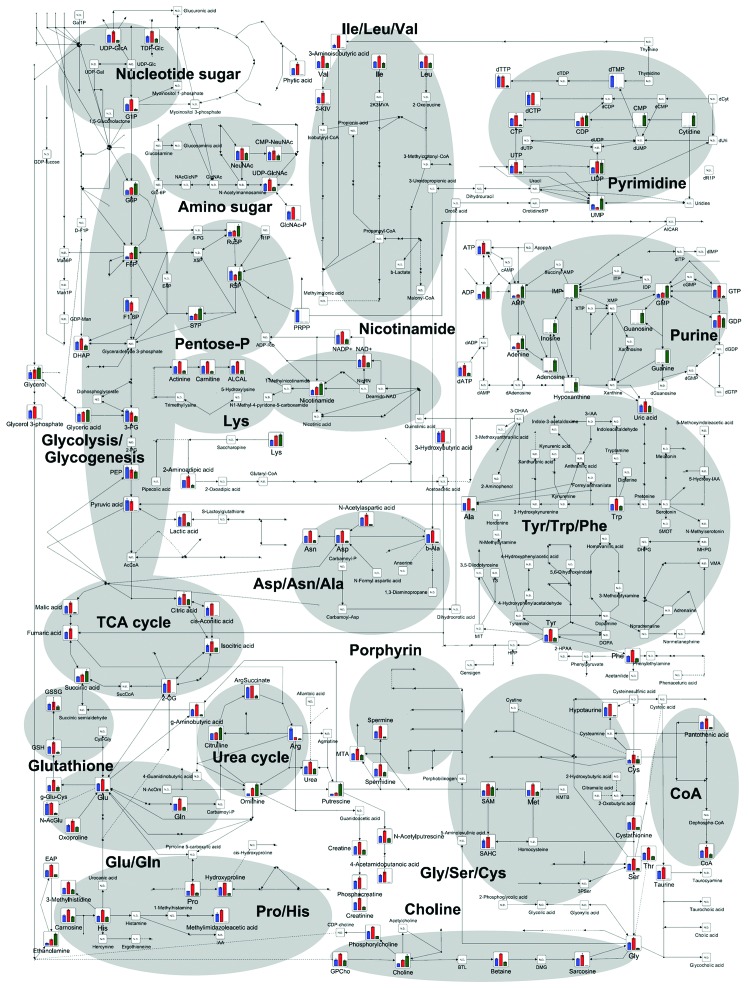
Metabolome data map of all metabolic pathways in HCT116 cells. Each bar represents the relative amount of a metabolite for non-treatment (blue) and treatment with FF/CAP18 at 10 μg/ml (red) or 40 μg/ml (green). All metabolite data are shown as the mean of triplicate samples ± standard deviation.

**Figure 3:**
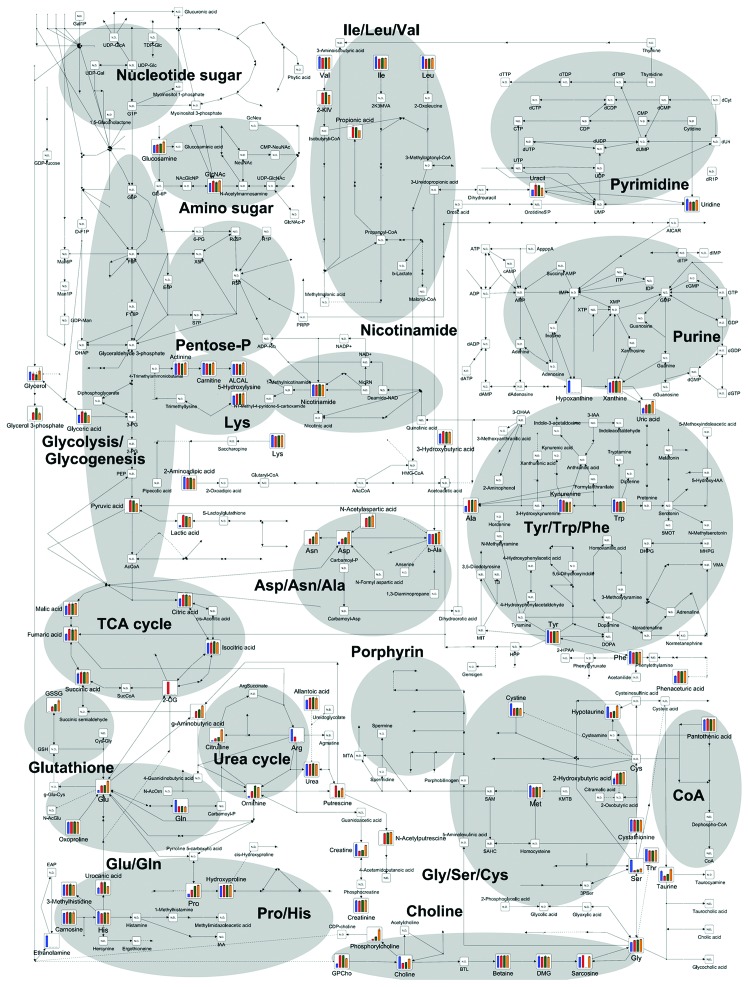
Metabolome data map of all metabolic pathways for conditioned medium. Each bar represents the relative amount of a metabolite for control medium (blue), conditioned medium after culture of HCT116 cells (red) and conditioned medium after culture of HCT116 cells treated with FF/CAP18 at 10 μg/ml (green) or 40 μg/ml (yellow). All metabolite data are shown as the mean of triplicate samples ± standard deviation.

**Figure 4:**
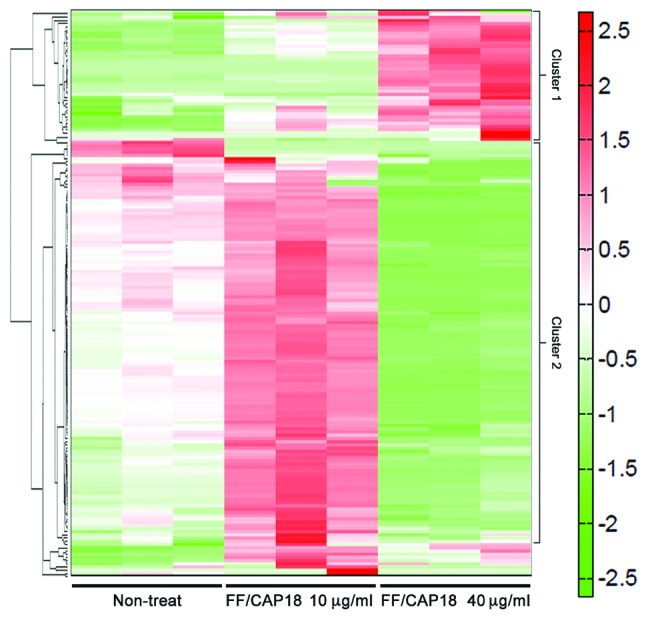
Euclidean-distance-based heat map of metabolites in non-treated HCT116 cells (left), and HCT116 cells treated with FF/CAP18 at 10 μg/ml (middle) or 40 μg/ml (right). The color scale from green to red indicates low to high correlation, respectively. Hierarchical cluster analysis was carried out using the proprietary software, PeakStat.

**Figure 5:**
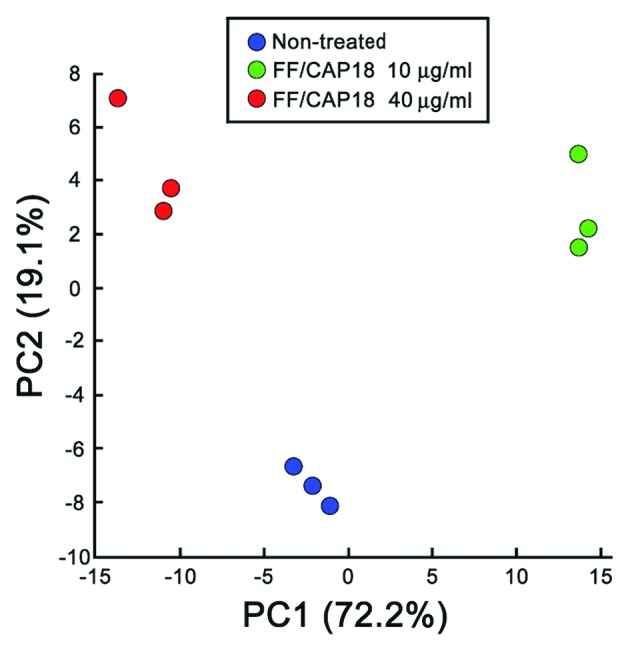
Principal component analysis (PCA) plot of metabolome analysis data that characterizes the trends exhibited by the expression profiles of non-treated HCT116 cells (blue), HCT116 cells treated with FF/CAP18 at 10 μg/ml (red) and HCT116 cells treated with FF/CAP18 at 40 μg/ml (green). The x-axis shows the distance of PC1 and the y-axis shows the distance of PC2 in the scatter plot. PCA was carried out using the proprietary software, SampleStat.

The 177 intracellular metabolites and 113 metabolites in conditioned medium were detected as peaks by CE-TOFMS, and mapped onto metabolic pathways for ease of viewing, as shown in Fig. 2 (cells) and Fig. 3 (conditioned medium). Overall trends of the intracellular metabolomic changes in HCT116 cells treated with FF/CAP18 and non-treated cells were analyzed by Euclidean-distance-based HCA, and the results are presented as a heat map (Fig. 4). The metabolomic profile of HCT116 cells treated with 10 μg/ml FF/CAP18 showed high values for the metabolites in cluster 2, including amino acids, and tricarboxylic acid (TCA) cycle intermediates. In contrast, the metabolomic profile of HCT116 cells treated with 40 μg/ml FF/CAP18 was reversed in comparison to the profile for treatment with 10 μg/ml FF/CAP18. We also confirmed that the metabolomic profiles of HCT116 cells treated with FF/CAP18 at 10 or 40 μg/ml were reversed for metabolites in cluster 1, including nucleotides and nucleosides. These trends were made even clearer from the results of the PCA of metabolome data for HCT116 cells treated with FF/CAP18 (Fig. 5). The concentration of FF/CAP18 was reflected in principal component 1; principal component 2 demonstrated the difference between treatment and non-treatment of cells with FF/CAP18. Therefore, treatment with FF/CAP18 exerted a dramatic change on the metabolism of HCT116 cells, and that change depended on the concentration of FF/CAP18.

### Purine metabolism

**Figure 6:**
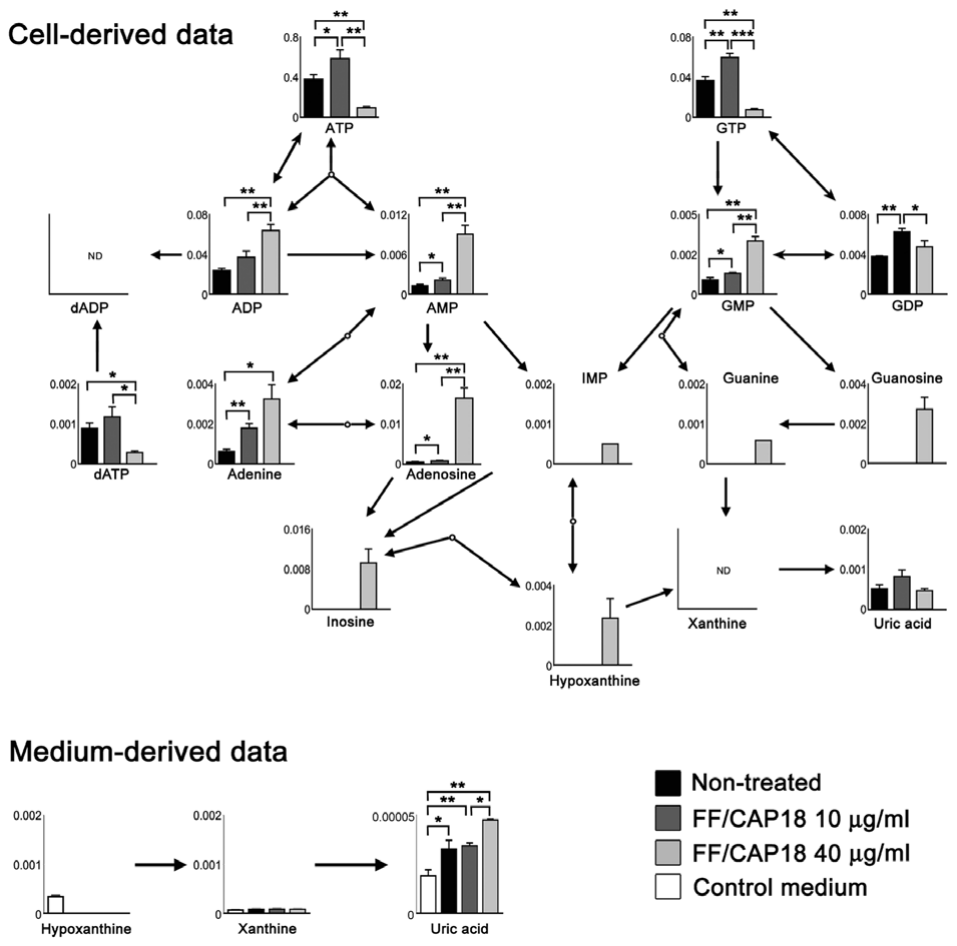
Metabolome data map of the purine metabolic pathway in HCT116 cells (cell-derived data) and conditioned medium (medium-derived data). Each bar represents the relative amount of a metabolite for non-treatment (black), treatment with FF/CAP18 at 10 μg/ml (deep grey) or 40 μg/ml (light grey) and control medium (white). All metabolite data are shown as the mean of triplicate samples ± standard deviation. The P-values were evaluated using Student’s t-test. The asterisks indicate the statistical significance. ^*^P<0.05; ^**^P<0.01; ^***^P<0.001.

Purine metabolism is an important pathway in order to supply nucleotides, such as 2′-deoxyadenosine triphosphate and 2′-deoxyguanosine triphosphate, for aggressive DNA synthesis in cancer cells. Fig. 6 shows the metabolic pathway of purine metabolism. The levels of adenosine triphosphate (ATP) and guanosine triphosphate (GTP) measured in HCT116 cells treated with FF/CAP18 at 10 μg/ml (dark grey bar) were higher than in non-treated cells (black bar); simultaneously, a slight increase was detected in the levels of adenosine diphosphate (ADP), adenosine monophosphate (AMP), guanosine monophosphate (GMP), and guanosine diphosphate (GDP). In contrast, in HCT116 cells treated with FF/CAP18 at 40 μg/ml (pale grey bar), there was a substantial decrease in the levels of ATP and GTP, whereas the levels of ADP, AMP, GMP, and GDP showed marked increases. These results suggest a high-energy status in HCT116 cells treated with the low dose (10 μg/ml) of FF/CAP18. However, HCT116 cells treated with the high dose of FF/CAP18 (40 μg/ml) fall into an energy-depleted status compared with non-treated and low-dose-treated cells. We could also confirm the presence of derivatives of purine, including adenine, hypoxanthine, and guanine, in HCT116 cells treated with a high dose of FF/CAP18. Additionally, increased uric acid was detected in conditioned medium sampled after culturing HCT116 cells with a high dose of FF/CAP18 (Fig. 6); uric acid is the final product of purine metabolism, and these data indicate that treatment of HCT116 cells with a high dose of FF/CAP18 facilitates the degradation of purine metabolites.

### Glycolysis and the pentose phosphate pathway (PPP)

**Figure 7:**
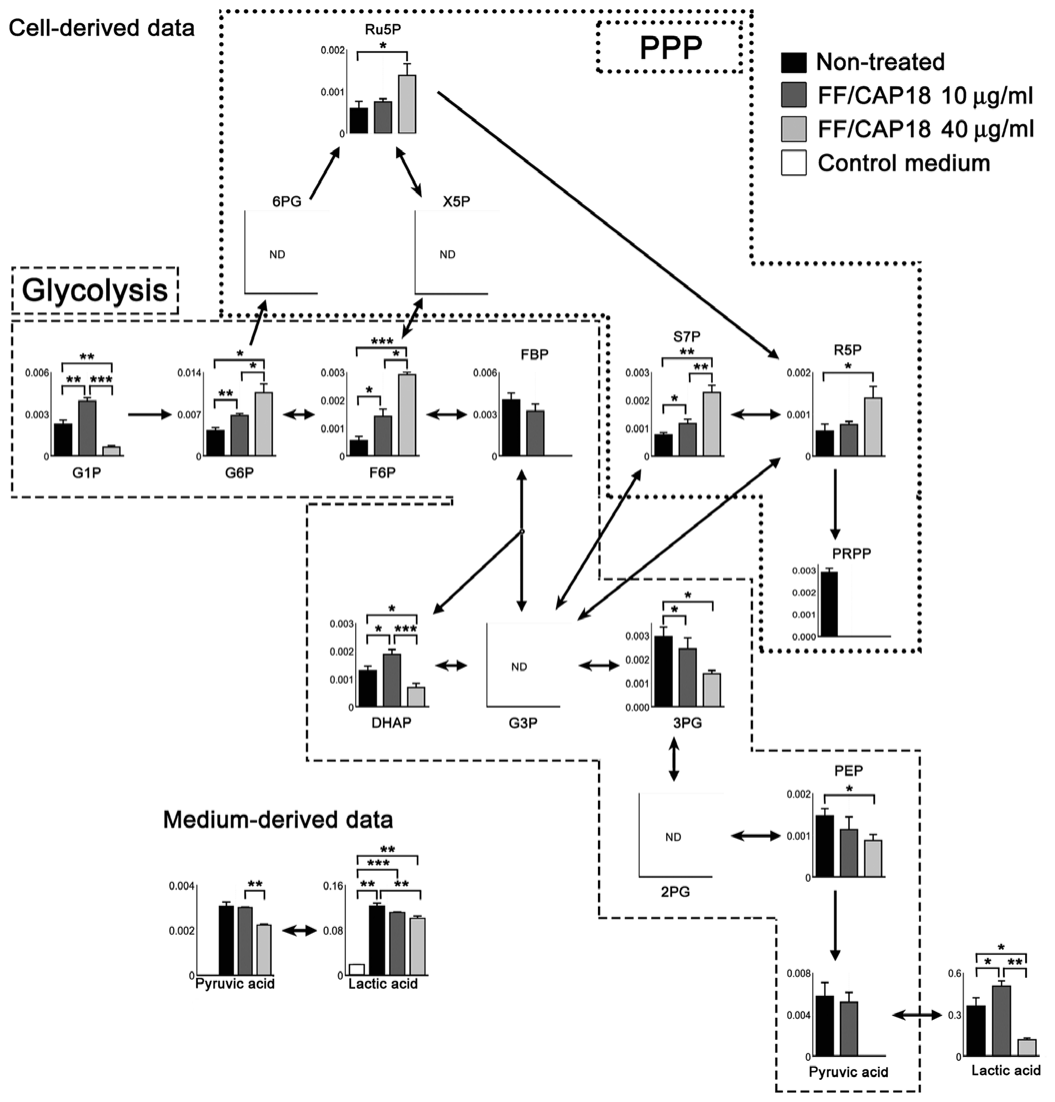
Metabolome data map of the glycolysis metabolic pathway and PPP, in HCT116 cells and in conditioned medium (medium data). Each bar represents the relative amount of a metabolite for non-treatment (black), treatment with FF/CAP18 at 10 μg/ml (deep grey) or 40 μg/ml (light grey) and control medium (white). All metabolite data are shown as the mean of triplicate samples ± standard deviation. The P-values were evaluated using Student’s t-test. The asterisks indicate the statistical significance. ^*^P<0.05; ^**^P<0.01; ^***^P<0.001.

Glycolysis is the key pathway for cancer cells to generate the energy that is required to support rapid cell division and cancer progression. We mapped the glycolysis pathway and a metabolic pathway branching from glycolysis, the PPP, as shown in Fig. 7. The levels of glucose 6-phosphate (G6P) and frucutose 6-phosphate (F6P) were increased after treatment with FF/CAP18, in a dose-dependent manner. The level of fructose 1,6-bisphosphate (FBP), however, tended to be lower in cells treated with 10 μg/ml FF/CAP18 than in non-treated cells, and FBP was not detectable in cells treated with FF/CAP18 at 40 μg/ml. Downstream metabolites, such as 3-phosphoglycerate, phosphoenolpyruvate, pyruvic acid and lactic acid similarly showed downregulation. Pyruvic acid and lactic acid in conditioned medium also showed a slight decline in level in HCT116 cells treated with FF/CAP18 (Fig. 7).

The PPP metabolites ribulose 5-phosphate (Ru5P), ribose 5-phosphate (R5P), and sedoheptose 7-phosphate (S7P) were upregulated in a dose-dependent manner, whereas 6-phosphogluconate and xylurose 5-phosphate were not detected. These data indicate that glycolysis in HCT116 cells was suppressed downstream of FBP in the presence of FF/CAP18, and the cells had shifted their metabolism towards the PPP.

### TCA cycle

**Figure 8:**
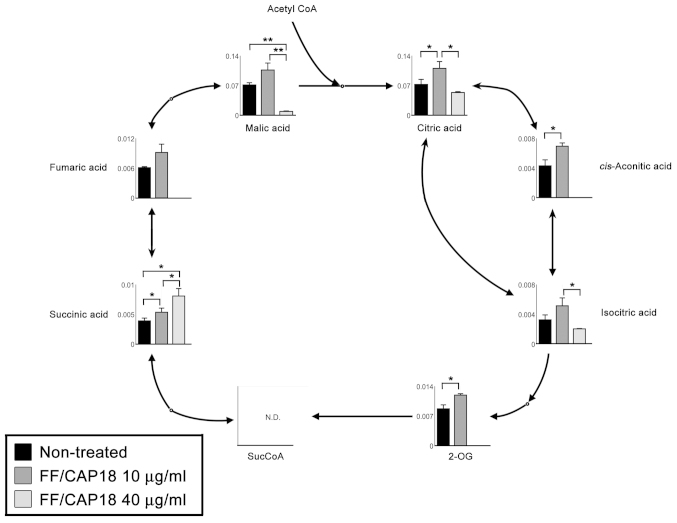
Metabolome data map of the TCA cycle in HCT116 cells. Each bar represents the relative amount of a metabolite for non-treatment (black), treatment with FF/CAP18 at 10 μg/ml (deep grey) or 40 μg/ml (light grey), and control medium (white). All metabolite data are shown as the mean of triplicate samples ± standard deviation. The P-values were evaluated using Student’s t-test. The asterisks indicate the statistical significance. ^*^P<0.05; ^**^P<0.01; ^***^P<0.001.

We observed significant differences in the levels of metabolites of the TCA cycle. While all metabolites detected in this study were upregulated in HCT116 cells treated with 10 μg/ml FF/CAP18, most metabolites of the TCA cycle were downregulated, with the exception of succinic acid, and then only in HCT116 cells treated with 40 μg/ml FF/CAP18 (Fig. 8); we could not confirm significant differences between HCT116 cells treated with FF/CAP18 at 10 and 40 μg/ml in the data for conditioned medium (Fig. 3). Therefore, the TCA cycle in HCT116 cells was likely to be facilitated by the administration of FF/CAP18 at 10 μg/ml. On the other hand, inactivation of the TCA cycle was induced through the accumulation of succinic acid.

## Discussion

Several studies have revealed anticancer activity of antimicrobial peptides, as demonstrated by oncolytic properties against many cancer cell types (26). However, the impact of antimicrobial peptides on cell metabolism is poorly understood, although metabolic reprogramming is a hallmark of cancer cells. Our study demonstrates large-scale metabolic profiling of colon cancer cells after treatment with an antimicrobial peptide, FF/CAP18, and suggests that the apoptotic cell death of colon cancer cells induced by FF/CAP18 is a result of dynamic levels of metabolites.

In the present study, PCA and HCA of large-scale metabolic profiling demonstrated remarkable differences between non-treated HCT116 cells, cells treated with FF/CAP18 at 10 μg/ml, and cells treated with 40 μg/ml FF/CAP18 (Figs. 4 and 5). A great number of metabolites, including nucleotides, TCA cycle components, and amino acids in HCT116 cells, increased in HCT116 cells after treatment with FF/CAP18 at 10 μg/ml compared with non-treated cells, indicating that FF/CAP18 could induce favorable metabolic conditions at this comparatively low concentration. On the other hand, at a concentration of 40 μg/ml, FF/CAP18 caused shortages of a number of metabolites. In previous studies, including our own, the cytotoxicity of various antimicrobial peptides towards cancer cells was reported to be dose-dependent (18,19,27). Thus, the results of the present study indicate that the levels of metabolites in cells undergoing apoptotic cell death caused by antimicrobial peptides do not show unidirectional movement in the same way as the concentration-dependent effects previously reported.

ATP generation occurs via glycolysis rather than oxidative phosphorylation in cancer cells; defined as the Warburg effect, this is a well-established metabolic characteristic of cancer cells. We observed upregulation of the first three intermediates in the glycolysis pathway [glucose 1-phosphate (G1P), G6P, and F6P], or of only the second two intermediates (G6P and F6P), after treatment of FF/CAP18 at 10 or 40 μg/ml, respectively (Fig. 7). However, as the levels of intermediates downstream of FBP were not increased in this dose-dependent manner, conversion of F6P to FBP is the rate-limiting step of glycolysis, and ATP generation via glycolysis is suppressed by administration of FF/CAP18. When apoptosis is induced in cells, their metabolism is regulated by various factors, such as the TP53-inducible glycolysis and apoptosis regulator, which blocks glycolysis at the stage of conversion of F6P to FBP and promotes activity of the PPP (28). G1P and fructose 1-phosphate are used to synthesize R5P, an intermediate of the PPP, as an alternative route for glucose metabolism. In this study, the levels of PPP intermediates (Ru5P, R5P, and S7P) were increased in FF/CAP18-treated HCT116 cells compared with non-treated cells (Fig. 7). Therefore, an antimicrobial peptide, FF/CAP18, can shift glucose metabolism towards the PPP and reduce the Warburg effect, resulting in slow glycolysis.

Interestingly, intermediates in the TCA cycle were increased by administration of FF/CAP18 at 10 μg/ml (Fig. 8), indicating that HCT116 cells acquire ATP via mitochondrial respiration, not glycolysis, in the early stage of apoptosis caused by FF/CAP18 treatment. On the other hand, most of the TCA cycle intermediates in HCT116 cells treated with FF/CAP18 at 40 μg/ml were downregulated (Fig. 8). This inverse relationship with the concentration of FF/CAP18 occurred in connection with the progression of apoptosis in treated HCT116 cells. Mitochondrial depolarization is a major event in the progression of apoptosis, and we confirmed that treatment with FF/CAP18 at 40 μg/ml induced this process in HCT116 cells in a previous study (19), suggesting that down-regulation of intermediates in the TCA cycle is potentially caused by this alteration in mitochondrial function.

Apoptotic cell death requires ATP for the progression of several steps, such as caspase activation, enzymatic hydrolysis of macromolecules (29,30), chromatin condensation (31), bleb formation (32), and the formation of apoptotic bodies (33). Zamaraeva *et al* suggested that elevation of the ATP level is a prerequisite for the apoptotic cell death process (34). FF/CAP18 treatment at 10 μg/ml could trigger apoptosis of HCT116 cells via upregulation of ATP generated by oxidative phosphorylation rather than glycolysis. Moreover, increasing the FF/CAP18 concentration to 40 μg/ml induced late-stage apoptosis that was accompanied by a reduction of the ATP level via suppression of glycolysis and the TCA cycle and resulted in conditions wherein most metabolites were depleted.

Owing to screening and/or improved treatment, colorectal cancer mortality rates have been observed to be decreasing in a large number of countries (35). However, the increasing prevalence of obesity and decreasing levels of physical activity in many parts of the world continue to contribute to the incidence of colorectal cancer. In addition, sensitivity to chemotherapy and severe side effects remain unresolved issues. Therefore, the discovery of novel therapeutic strategies for colorectal cancer is the focus of intense research efforts.

Antimicrobial peptides are an essential component of the innate immunity of many organisms, and target a wide-range of infectious disease agents, such as fungi, protozoa (36), the human immunodeficiency virus and herpes viruses (37). Whereas antimicrobial peptides have been studied as antimicrobial agents, their potential as anticancer peptides in cancer therapy, either alone or in combination with other conventional drugs, has been regarded as a therapeutic strategy yet to be explored (26). Indeed, several recent studies have suggested that antimicrobial peptides possibly exert cytotoxic effects against colon cancer via apoptotic death (17,19,38). Thus, our study, approaching this issue from the point of view of metabolic changes, can support an understanding of the mechanisms of anticancer agents such as antimicrobial peptides, which can in turn facilitate the discovery of novel remedies or therapeutic strategies for cancer.
